# Treatment of Severe Pulmonary Regurgitation in Enlarged Native Right Ventricular Outflow Tracts: Transcatheter Pulmonary Valve Replacement with Three-Dimensional Printing Guidance

**DOI:** 10.3390/bioengineering10101136

**Published:** 2023-09-28

**Authors:** Yiwei Wang, Ping Jin, Xin Meng, Lanlan Li, Yu Mao, Minwen Zheng, Liwen Liu, Yang Liu, Jian Yang

**Affiliations:** 1 Department of Cardiovascular Surgery, Xijing Hospital, Air Force Medical University, Xi’an 710032, China; wangyiwei0911@hotmail.com (Y.W.); bohua001@126.com (P.J.);; 2 Department of Ultrasound Medicine, Xijing Hospital, Air Force Medical University, Xi’an 710032, China; 3 Department of Radiology, Xijing Hospital, Air Force Medical University, Xi’an 710032, China

**Keywords:** transcatheter pulmonary valve replacement, pulmonary regurgitation, enlarged native right ventricular outflow tract, PT-Valve, three-dimensional printing

## Abstract

Background: Severe pulmonary regurgitation (PR) often occurs following a transannular patch repair of tetralogy of Fallot, resulting in an enlarged native right ventricular outflow tract (nRVOT) with varying shapes. Methods: We conducted a single-center study with eight patients having severe PR and enlarged nRVOT (diameters ≥ 29 mm). Transcatheter pulmonary valve replacement (TPVR) was performed using the self-expanding PT-Valve. Preoperative evaluation included echocardiography, computed tomography, and magnetic resonance imaging. A 3D-printed model of the nRVOT was used for preoperative assessment. Follow-up data were collected in 1-year follow-up. Results: PT-Valve was successfully implanted in all patients, resulting in immediate improvement of severe PR. Pulmonary artery diastolic pressure increased significantly (*p* < 0.001). No deaths or coronary compression occurred during the procedure. Over a 1-year follow-up, no stent displacement or fracture occurred. Only two patients had trace paravalvular leaks. Magnetic resonance imaging revealed a reversal of right ventricular remodeling, with a significant reduction in right ventricular end-diastolic volume index (*p* < 0.001) and improved right ventricular ejection fraction (*p* < 0.001). All patients achieved primary endpoints. Conclusion: 3D printing-guided PT-Valve implantation in enlarged nRVOT for severe PR is safe and effective, expanding TPVR indications and offering potential treatment for a broader patient population.

## 1. Introduction

Severe pulmonary regurgitation (PR) is the commonly encountered lesion following repair of pediatric and adult tetralogy of Fallot (TOF), and the patients eventually end up with right ventricular dilation and dysfunction, arrhythmia, and limited exercise capacity [1,2,3]. Transcatheter pulmonary valve replacement (TPVR) has been validated as a promising therapeutic option for PR, reducing the need for additional surgical interventions in selected patients [3,4,5,6,7,8]. However, the currently widely used balloon-expandable systems for TPVR, namely the Melody (Medtronic, Minneapolis, MN, USA) and the SAPIEN platforms (Edwards Lifesciences, Irvine, CA, USA), are only recommended for implantation in designated landing zones, which are homografts or conduits forming the right ventricular outflow tract (RVOT) [4,5,6,8]. Unfortunately, approximately 80% of patients who undergo surgical RVOT reconstruction receive either a valve-sparing repair or a transannular patch [1,2,9]. This treatment not only results in severe PR at a later stage but also leads to an enlarged native RVOT (nRVOT). Moreover, the availability of types and sizes of percutaneous valves for these patients is limited [3,4,5,6,8,9].

Several techniques and devices are currently being developed to address these challenges. These techniques include the use of a “Russian doll” approach, where multiple stents are overlapped to reduce the diameter of the RVOT. Another technique, known as “pulmonary jailing,” involves utilizing a pulmonary artery branch to create a secure landing zone within the RVOT [4,5,6]. Additionally, ongoing research on dedicated transcatheter pulmonary valves, such as the Harmony valve (Medtronic, Minneapolis, MN, USA) and the Venus *p*-valve (MedTech, Shanghai, China), has demonstrated promising outcomes [10,11]. Given the complex nature of the enlarged nRVOT and the associated risks of complications such as coronary artery compression, valve migration, and embolization, it is essential to conduct comprehensive preoperative assessments and simulations [12,13]. Engaging in these steps will help determine the feasibility of the devices and ensure the success of the procedure [12,13,14].

Cardiovascular three-dimensional (3D) printing technology is extensively used in various applications, particularly for providing surgical guidance in cases of congenital and structural heart diseases [12,13,14,15,16]. It has proven to be an effective tool for evaluating the potential occurrence of associated complications. Additionally, this technology can be utilized to investigate the distinctive characteristics and properties of novel devices [15,16]. Our goal was to report the single-center experience with TPVR to treat patients with severe PR and an enlarged nRVOT utilizing a novel dedicated device, the PT-Valve (Beijing Med-Zenith, Beijing, China). Additionally, we explored the guiding role of 3D printing in simulating the main procedures and evaluating potential risks associated with TPVR.

## 2. Materials and Methods

### 2.1. Study Population

The study was a single-center, observational study. From February 2022 to August 2022, a total of eight patients with severe PR and enlarged nRVOT following the surgical repair of TOF [four females; 17.0 (12.0, 52.0) years] were enrolled in this study. The inclusion criteria were as follows: (1) patients who were at least 10 years old or weighed more than 25 kg; (2) PR fraction ≥ 30% confirmed by cardiac magnetic resonance imaging (MRI) or a severity grade of PR ≥ 3+ measured by transthoracic echocardiography; (3) New York Heart Association (NYHA) functional class ≥ II or = I; and concomitant right ventricular end-diastolic volume index (RVEDVI) ≥150 mL/m^2^ recommended by the 2020 European Society of Cardiology guidelines for the management of adult congenital heart disease [17]. (4) A diameter of the nRVOT > 29 mm. The exclusion criteria were as follows: (1) mean pulmonary artery (PA) pressure ≥ 30 mmHg revealed by right heart catheterization; (2) preexisting or active infective endocarditis; (3) acute decompensated heart failure; (4) the narrowest plane for the right ventricle–PA (RV–PA) conduits with a diameter of 40 mm or more; (5) prior mechanical tricuspid valve replacement or insufficient diameter of the femoral vein for introduction of a delivery system. This study was reviewed and approved by the Xijing Hospital Ethics Committee (KY20192138-C1) and conducted in accordance with the principles of the Declaration of Helsinki. The ethical registration was entered in the ClinicalTrials.gov Protocol Registration System (NCT02917980; 27 September 2016). All of the aforementioned work has been conducted within the realm of structural heart disease, with a primary focus on valvular heart disease.

### 2.2. Preprocedural Imaging

Gated cardiac computed tomography and MRI were utilized to assess the anatomical structures and determine the potential for coronary artery compression. Invasive right heart catheterization was used to evaluate the hemodynamics of the right ventricle (RV) and the PA. Additionally, both transthoracic echocardiography and transesophageal echocardiography (TEE) were conducted on all patients prior to the procedures to assess the function of the pulmonary valve and the RVOT.

### 2.3. Device Description

**Figure 1:**
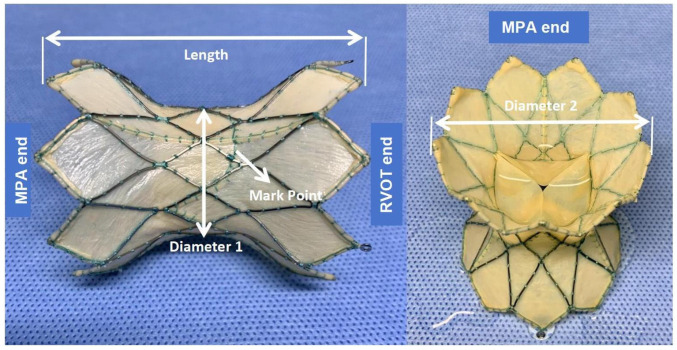
PT-Valve sizing. The PT-Valve is a symmetrical, dumbbell-shaped bioprosthesis with five different sizes utilized. Diameter 1 is the porcine valve size in the middle of the frame with 20, 23, and 26 mm, respectively. Diameter 2 represents the outflow and inflow diameters of the valve frame, which are equal and come in sizes of 28, 32, 36, 40, and 44 mm, respectively. There are three Mark points at the base of the valve leaflet to indicate the position of the leaflet intraoperatively. The length of the frame has five sizes: 38, 42, 46, 50, and 54 mm to fit the conduits. MPA—main pulmonary artery; RVOT—right ventricular outflow tract.

The PT-Valve (Beijing Med-Zenith, Beijing, China) is a pericardial tissue valve derived from porcine sources. It is mounted on a self-expanding nitinol frame that is covered by porcine pericardium. The valve frame is available in five different sizes to accommodate the various morphologies of nRVOT. The nitinol frame is laser-cut and designed with a symmetrical shape to ensure stability and a tight seal on both sides of the RV–PA conduit. The outflow and inflow diameters of the valve frame are equal and come in sizes of 28, 32, 36, 40, and 44 mm, respectively. The length of the frame ranges from 38 to 54 mm, and the porcine valve itself has diameters of 20, 23, and 26 mm in the middle of the frame. It is intentionally smaller than the outflow and inflow diameters of the frame to avoid compression. To mitigate leaflet calcification, the tissue prosthetic is pretreated with specific “alcohol” and “surfactant” (Figure 1).

The delivery system (21F) comprises an outer sheath that compresses the stent, an attachment system featuring three recessed areas for the placement of corresponding stent extenders, and a short-tapered nose cone to aid in smooth delivery. Additionally, a loading attachment has been developed to assist in folding the stent within the outer sheath. To ensure accurate positioning, a manually controlled release system allows for a gradual withdrawal of the outer sheath, starting from the distal frame and controlling stent expansion during device delivery.

### 2.4. 3D Reconstruction and Preprocedural 3D Testing In Vitro

**Figure 2:**
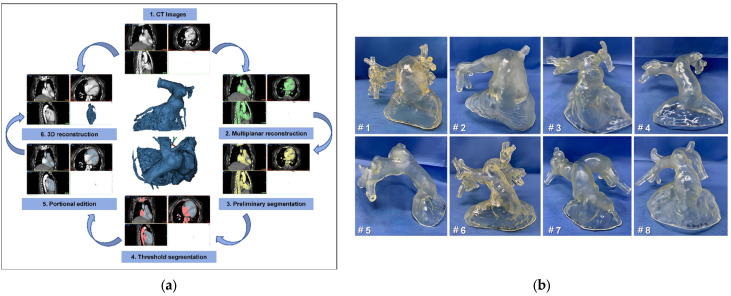
Preoperative three-dimensional (3D) reconstruction and enlarged right ventricular outflow tract 3D-printed models to help surgeons understand anatomical structures and make an accurate surgical plan. (**a**) The process of 3D reconstruction using Materialise Mimics version 21.0 (Materialise, Leuven, Belgium). (**b**) The 3D-printed models restore the enlarged native right ventricular outflow tracts and pyramidal right ventricle–pulmonary artery conduits of all eight patients; #1–#8, patient 1–8.

The patients’ computed tomography angiography (CTA) data were imported into Materialize Mimics 21.0 version software (Leuven, Belgium). Three orthogonal sections (coronal plane, sagittal plane, and cross-plane) were established using the multiplane interactive reconstructed imaging function of the software based on actual needs. Then, the initial digital 3D model of the RV–PA conduit and the adjacent tissues reconstruction were reconstructed by repeated comparisons and confirmations, and the collected data were converted to the standard format of Digital Imaging and Communication of Medicine for storage. The comprehensive reconstruction of the RV–PA and the distal PA morphology was performed using Materialise 3-matic software (Materialise, Leuven, Belgium). In addition, different parts of the digital 3D reconstructed model were exported into the Standard Tessellation Language format, and the resulting files were imported into the Polyjet 850 multi-material full-color 3D printer (Stratasys, Inc., Eden Prairie, MN, USA) for printing to produce the patient-specific 3D model (Figure 2). We chose to base the patient-specific 3D-printed model on CTA data from the end-systolic phase, characterized by minimal amplitude cardiac motion and, accordingly, by the least expected blurring due to motion artifacts. More importantly, using the CTA data ensures the production of the largest diameter of nRVOT and RV–PA conduits and that the optimal size of the implanted device is determined during simulation using the 3D-printed model, minimizing complications.

**Figure 3:**
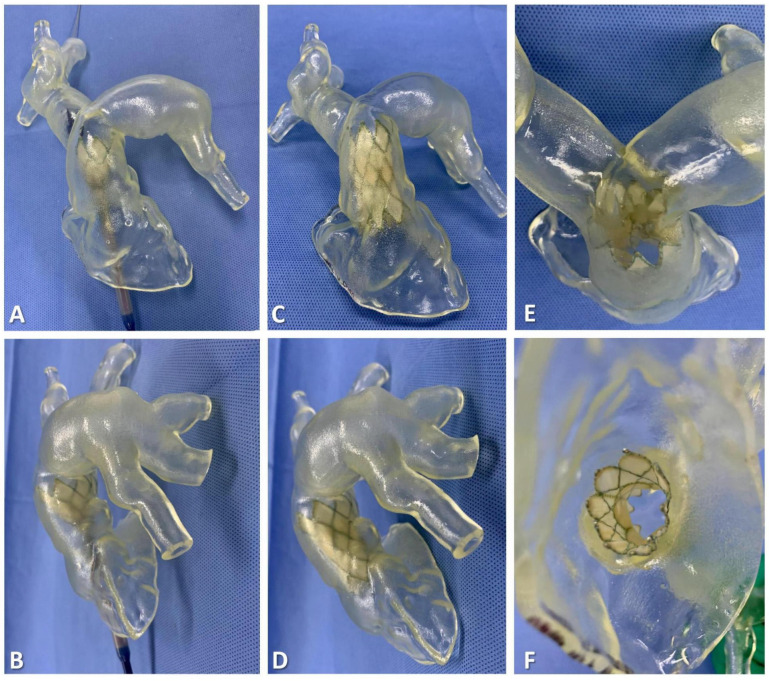
Simulation of transcatheter pulmonary valve replacement using a three-dimensional printed model for the bench test (e.g., data for patient #7). (**A**,**B**) The delivery system was inserted into the right ventricle–pulmonary artery conduit, and then the bioprosthesis was released for positing in the distal landing zone. (**C**,**D**) When the bioprosthesis is fully released, and the delivery system is withdrawn, the stability can be evaluated. (**E**,**F**) The stent effectively seals both ends of the conduit, ensuring there is no paravalvular leak.

**Figure 4:**
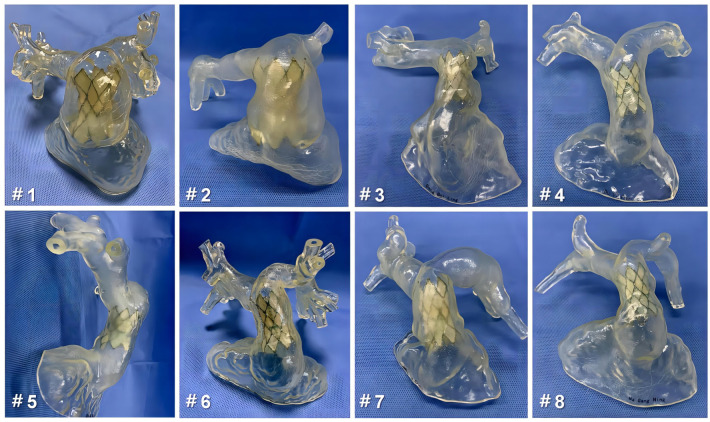
The implantation of PT-Valve using 3D-printed models of all eight patients predicts the morphology and functionality of the bioprostheses without paravalvular leak and stent displacement. To minimize the risk of coronary compression, there is no contact between the conduit and the middle of the stent.

The guidewire was carefully inserted through the PA branch of the 3D-printed model, eventually exiting the RVOT. With the guidance of the guidewire, the delivery system was successfully maneuvered into the RV–PA conduit. Taking into account the complex shapes and sizes of the conduit, the appropriate distal landing zone for the stent was selected. Following the release of the stent, close attention was given to assessing the anchoring planes at both ends of the stent and ensuring its stability. This evaluation was crucial in determining the risk of a paravalvular leak (PVL) and potential stent compression. By carefully monitoring these factors, any concerns or issues could be promptly identified and addressed to ensure optimal performance and functionality of the implanted device (Figure 3 and Figure 4).

### 2.5. Procedural Steps

**Figure 5:**
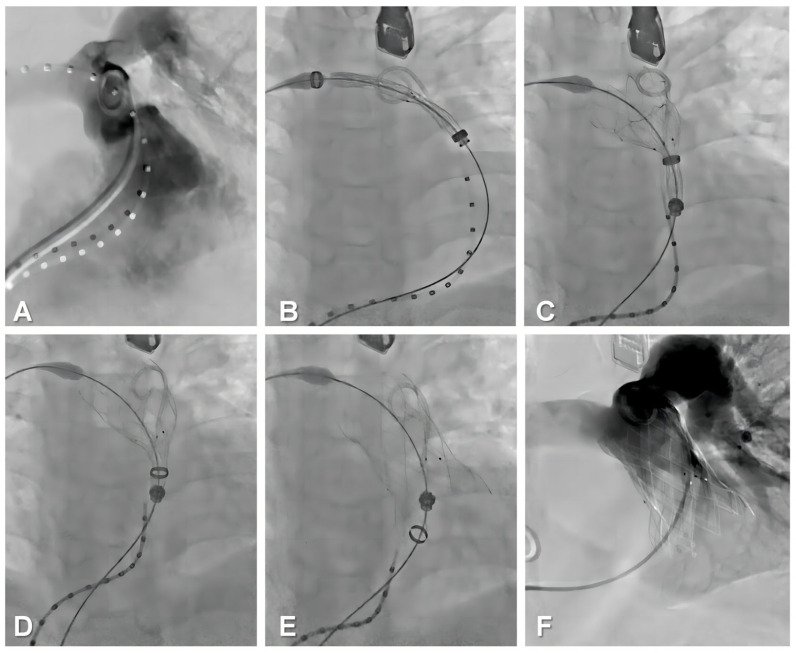
The implantation of a PT-Valve for the transcatheter pulmonary valve replacement procedure. (**A**) Fluoroscopy imaging revealed severe pulmonary regurgitation and an enlarged native right ventricular outflow tract. (**B**) The distal valve-containing capsule was transported to the predetermined position and confirmed under fluoroscopy imaging. (**C**) Outflow portions of the device were released. (**D**) The mid-segment of the device was released. (**E**) Inflow portions of the valve were finally released. (**F**) Fluoroscopy imaging showed that pulmonary regurgitation disappeared immediately.

All procedures were performed with the patient under general anesthesia. The right femoral vein was punctured for implantation of the delivery system (21 F). Then, the angiographic catheter was inserted via the left femoral vein into the RV to present the position and geometry of the RV–PA and to guide precise implantation of the bioprosthesis. Given the dumbbell shape, the slender waist of the frame design, and the preprocedural 3D reconstruction and 3D printing guidance, a coronary artery compression test was not performed before the bioprosthesis was implanted. The delivery system was introduced to the RV–PA after a Double Curve Lunderquist guidewire (Cook Medical, Bloomington, IN, USA) was placed in the distal PA. Then, the appropriately sized stent was deployed in the selected landing zone according to the evaluation and simulation of preprocedural 3D printing guidance. After performing angiography to rule out PVL and PR and confirming excellent hemodynamics using TEE, the delivery system was withdrawn and removed, and the femoral venous puncture points were closed surgically with sutures (Figure 5).

### 2.6. Data Collection

Baseline data were collected from the electronic medical record system. The operating time and the fluoroscopy time were recorded. In addition, data were collected during the hospitalization period (including TEE data obtained immediately postoperatively and the time in the intensive care unit and in the hospital).

### 2.7. Follow-Up

The data were collected from enrolled patients at various time points: at baseline, before discharge, and at 30 days, 6 months, and 1 year after the operation. The primary endpoints of the study were to achieve a successful operation and to implant the device successfully. A successful valve implant was defined as follows: The valve was placed securely in the desired position, and the bioprosthesis functioned satisfactorily [PR severity was reduced to ≤mild, the catheterized peak RV–PA gradient (PG_max_) ≤ 35 mmHg], without the need for device removal within 24 h of implantation. The secondary goals of the study were to observe if there were any cardiovascular-related deaths, implant displacements, valve failures, or other major adverse events associated with the device. These adverse events included valve embolism, stent fracture, structural deterioration (excluding stent fracture), PVL, thrombosis, pulmonary embolism, endocarditis, and hemolysis.

### 2.8. Statistical Analyses

Continuous variables were reported as the median with the 25th and 75th percentiles; the categorical variables were presented as frequencies and percentages. A two-tailed *p*-value of less than 0.05 was considered statistically significant. All statistical analyses were performed using Statistical Package for Social Sciences (SPSS, Chicago, IL, USA) version 26.0.

## 3. Results

### 3.1. Baseline Data

The baseline clinical characteristics are presented in Table 1. A total of eight patients with TOF were enrolled in this study; four were males. Among the enrolled patients, six underwent surgical RVOT reconstruction with a transannular patch, whereas the remaining two underwent pulmonary atresia repair. The mean age of the patients was 25.8 years (range: 12–52 years), and their mean body weight was 61 kg (range: 50–75 kg). Five patients were classified as NYHA functional class III–IV, whereas three patients were classified as NYHA functional class II. The mean distance of the 6-min Walk Test, which was 348.8 m (range: 290–420 m), demonstrated decreased exercise capacity in eight patients.

The baseline imaging parameters are presented in Table 2. Among the enrolled patients, eight had severe (grade 4+) PR, with five of them also exhibiting moderate to severe (3+/4+) tricuspid regurgitation. The mean peak transpulmonary valve gradient, as measured by echocardiography, was 28.8 mmHg (range: 21–35 mmHg). Impaired RV function was indicated by the tricuspid annular plane systolic excursion, which had a mean of 15.9 mm (range: 15–18), and by the RV fractional area change, which had a mean of 33.3% (range: 31–36). The CTA images revealed various morphologies of RV–PA conduits. The mean length of the RV–PA conduit was 52.5 mm (range: 43–63), and all patients showed an enlarged nRVOT, with a mean diameter of 35 mm (range: 30–46), exceeding 29 mm in all cases. The mean narrowest plane diameter of the RV–PA conduit was 26.6 mm (range: 22–37), with three cases located at the distal main pulmonary artery (MPA), three at the mid-MPA, and two at the pulmonary annulus. MRI images exhibited signs of RV dysfunction, as indicated by the RVEDVI [mean 166.8 mL/m^2^ (range: 156–189)] and the right ventricular ejection fraction [mean 47% (range: 40–56)]. The mean PR fraction was 49.4% (range: 40–59).

Based on the guidance provided by preoperative 3D-printing technology, patients 1 and 2 were fitted with 44–26 mm devices, patients 3, 7, and 8 received 36–23 mm devices, and patients 4, 5, and 6 were given 32–23 mm devices. Table 2 presents the optimal landing zones selected through simulation using patient-specific 3D-printed models.

### 3.2. Perioperative and In-Hospital Outcomes

The perioperative and in-hospital outcomes are summarized in Table 3. Devices were successfully implanted in all patients, with sizes and landing zones determined based on preoperative 3D printing guidance. Each patient received a single device. The mean procedure time was 61.8 min (range: 52–71), and the mean fluoroscopy time was 19 min (range: 14–26). No instances of device malposition, coronary compression, or reduced flow to the PA branches were observed during the procedures. After the procedure, the right heart catheterization parameters showed no significant change in PA systolic pressure [from 35.5 (32, 36) mmHg to 37 (35, 38) mmHg, *p* = 0.762], but the PA diastolic pressure increased from 3.5 (3, 6) mmHg to 10 (8, 11) mmHg (*p* < 0.001). The peak transpulmonary valve gradient was decreased from 28.5 (26, 33) mmHg to 8.5 (7, 10) mmHg (*p*< 0.001) after the deployed device was viewed by TEE. Following the procedure, right heart catheterization parameters showed no significant change in PA systolic pressure [from 35.5 (32, 36) mmHg to 37 (35, 38) mmHg, *p* = 0.654]. However, there was an increase in PA diastolic pressure from 3.5 (3, 6) mmHg to 10 (8, 11) mmHg (*p* < 0.001). No ventricular arrhythmias, device-related adverse events, regurgitation, or PVL above grade 2+ (≥mild) occurred following valve deployment. Two patients experienced minor postoperative hemoptysis, likely attributed to the sudden increase in PA pressure following device implantation and subsequent capillary rupture. However, all patients successfully recovered from this condition and were discharged from the intensive care unit within 2 days. Before discharge, the CTA data of patients were utilized for 3D reconstruction, and 3D models were printed to validate the postoperative morphology and functionality of the bioprostheses (Figure 5). Prophylactic antibiotics were administered for a standard duration of 3 days following the procedure.

### 3.3. One-Year Follow-Up Data

**Figure 6:**
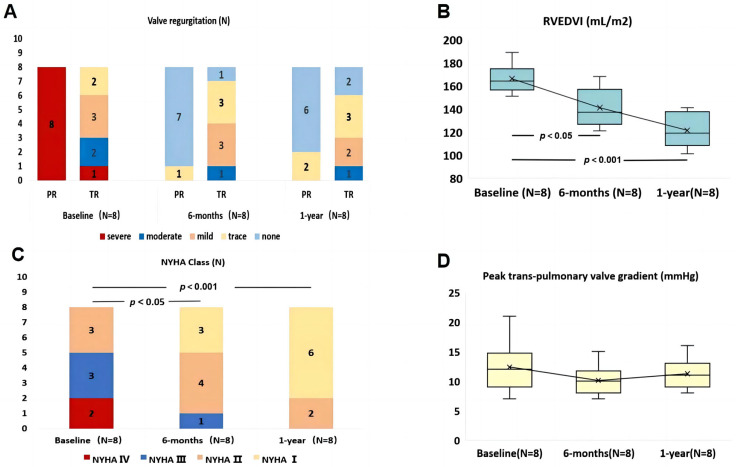
Follow-up data after PT-Valve implantation. (**A**) pulmonary regurgitation and tricuspid regurgitation severity; (**B**) right ventricular ejection fraction volume index; (**C**) New York Heart Association class; (**D**) peak trans-pulmonary valve gradient; NYHA—New York Heart Association; PR—pulmonary regurgitation; RVEDVI—right ventricular ejection fraction volume index; TR—tricuspid regurgitation.

The major follow-up outcomes at 1 year are presented in Table 4. Patients were prescribed dual antiplatelet therapy consisting of aspirin (100 mg/day) and clopidogrel (75 mg/day) for a period of 6 months. Subsequently, patients continued with aspirin monotherapy (100 mg/day) for a period of 1 year. The patients were followed up for a period ranging from 12 to 19 months, during which no instances of early valve decay or reintervention were observed. All patients underwent relevant examinations at 30 days, 6 months, and at the 1-year follow-up. Echocardiography examinations at the 1-year follow-up revealed that two patients exhibited grade 1 (trace) PVL without the need for intervention. The remaining patients did not experience any PR or PVL. The implanted devices demonstrated favorable performance, as evidenced by a decrease in the PR fraction [from 50 (41, 55)% at baseline to 1.5 (1, 2)% at 1 year (*p* < 0.001)]. MRI findings revealed a reversal of right ventricular remodeling, characterized by a significant reduction in the RVEDVI [from 164 (156, 176) mL/m^2^ at baseline to 119 (107,148) mL/m^2^ at the 1-year follow-up, respectively (*p* < 0.001)]. Consequently, there was an improvement in the right ventricular ejection fraction [from 22.5 (19, 26)% at baseline to 33 (31, 39)% at the 1-year follow-up, respectively (*p* < 0.001)], indicative of enhanced right ventricular function. Furthermore, there were continuous improvements in NYHA class, peak O_2_ levels, and 6-Minute Walk Test distances following the TPVR procedure, suggesting an enhancement in exercise capacity and overall quality of life (Figure 6).

## 4. Discussion

In this single-center observational study, the PT-Valve was successfully implanted in all eight patients with enlarged nRVOT (all diameters ≥ 29 mm), leading to favorable clinical outcomes for severe PR. In addition to routine imaging assessments, our preoperative guidance, utilizing cardiovascular 3D printing technology, facilitated the selection of appropriate device sizes and optimal implant sites by interventionalists.

Severe PR following surgical repair of TOF is a life-threatening condition that often presents with chronic overload, resulting in significant right ventricular dysfunction and clinical symptoms [1,2,9,18]. TPVR has shown promise as an alternative treatment for severe PR in patients with suitable anatomy, offering a favorable prognosis. However, the procedure remains challenging due to the variable and irregular morphology and size of the nRVOT and the RV–PA conduits following early TOF repair procedures (e.g., transannular patches) [7,8,9,14,15,16]. A study by Schievano et al. [19], which analyzed 83 patients who underwent RVOT reconstruction using cardiac MRI and 3D digital reconstruction, revealed that the RVOT anatomy could be classified into five visually recognizable patterns. However, significant differences were observed within each category, indicating the unpredictability and wide variability encountered in this complex patient population, with no direct correlation found between surgical history or underlying diagnosis and subsequent nRVOT morphological categorization. Furthermore, within the cardiac cycle, the diameter of the RVOT can vary by more than 50% in a single patient, exhibiting axial shortening and lengthening [20]. This dynamic nature of the structures adds to their complexity. Additionally, although TPVR is a minimally invasive procedure, it is not without associated complications, including stent fracture, valve embolization, PVL, and coronary compression [12,13,14,21,22]. To mitigate these potential complications and enhance the likelihood of procedural success, further research, continuous development, and improvement of new devices, as well as comprehensive preoperative studies of nRVOT anatomy, are essential [23]. As a result, interventional cardiologists are actively seeking new solutions.

Currently, the clinical use of balloon-expandable devices for TPVR is dominated primarily by two types: the Melody (Medtronic, Minneapolis, MN, USA) and the SAPIEN platforms (Edwards Lifesciences, Irvine, CA, USA). However, the indications for enlarged nRVOT are still largely restricted by the diameter of the annulus (up to 29 mm) and the challenge of achieving stable landings [4]. Prestenting is currently used to create a uniform artificial tube in irregularly shaped RV–PA conduits. This approach facilitates the secure and properly positioned implantation of cylindrical transcatheter heart valves, minimizing the risk of valve displacement and PVL. Fuente et al. [8] conducted TPVR using balloon-expandable valves following pre-stenting in a cohort of 46 patients with nRVOT conditions. Among these patients, five had a notably large nRVOT and a pyramidal RV–PA conduit. Although these cases demonstrated favorable clinical outcomes at the midterm follow-up, valve re-expansion was still required in some instances. In a multicenter study by Martin et al. [5], pre-stenting was performed for 229 patients with nRVOT. However, secondary catheterization was unsuccessful in 97 patients, primarily due to the excessively large size of the nRVOT and the potential risk of coronary compression.

These studies also underscore the importance of improved assessment of preoperative imaging data and the selection of appropriately sized devices, which significantly enhance the feasibility of TPVR in the context of an enlarged nRVOT. The pre-stenting approach, although effective, comes with certain drawbacks. It involves additional radiation exposure, leading to prolonged procedure times. Furthermore, a delay of several months in the secondary intervention of the prosthesis is necessary to allow for stent integration, ensuring stent stability [24]. However, this approach is susceptible to stent extrusion or folding during TPVR. Additionally, the population eligible for transcatheter valve therapy is evolving with the introduction of novel self-expanding transcatheter pulmonary valves. Among these valves is the Venus *p*-valve (Venus Medtech, Shanghai, China), which has demonstrated promising results in early clinical trials and has received the CE mark [11,25]. Similarly, the Harmony valve (Medtronic, Inc.) has also obtained FDA approval [10]. Other valves, such as the Pulsta valve (Tae Woong Medical Co, Gyeonggi-do, South Korea), are still undergoing clinical trials [25,26]. These new devices have the potential to expand the eligibility criteria for TPVR to include patients with an nRVOT width of up to 34 mm. However, it is crucial for these devices to be compatible with specific anatomical structures, such as the absence of significant funneling, trunk, or branching stenosis. This compatibility allows for the proper distal and proximal expansion of the self-expanding valve and ensures secure anchoring to the wall of the nRVOT.

Furthermore, cardiovascular 3D printing has found extensive applications not only in surgical guidance for congenital heart disease but also in the interventional treatment of structural heart disease, showcasing significant advancements [12,13,14,15,16]. Utilizing composite materials such as silicone, resin, and polyethylene with varying hardnesses, models can be printed to accurately replicate the anatomical structure of individual patients. Patient-specific 3D-printed models of enlarged nRVOTs offer valuable insights for improving device design concepts, exploring device features and feasibility, and facilitating preprocedural planning for TPVR. This approach leads to reduced radiation and contrast usage during fluoroscopy and yields improved procedural outcomes. 3D printing technology serves as an invaluable tool for replicating abnormal anatomy and facilitating more comprehensive preprocedural planning in cases of enlarged nRVOT for both interventions and observation of optimal landing zones after bioprosthesis implantation. In 2007, Silvia et al. [14] utilized 3D printing to assess the suitability of TPVR for their patients and demonstrated that 3D modeling allowed for more precise patient selection for TPVR compared to using MRI images as a procedural guide.

In another study, Shafkat et al. [13] employed flexible materials to create a 3D-printed model of a 15-year-old male who had previously undergone TOF repair with nRVOT dimensions at the marginal diameter. The patient underwent a pre-stenting implant simulation using the model, which demonstrated the feasibility of a catheter-based strategy. Subsequently, a TPVR procedure was performed based on the preoperative simulation plan, resulting in successful outcomes and obviating the need for surgery. Francesca et al. [12] utilized 3D-printed models to simulate and evaluate the steps of the “pulmonary jailing” procedure. They demonstrated that the selection of the appropriate cardiac cycle for printing and the adjustment of printing material properties are crucial when evaluating the outcomes. In conclusion, cardiovascular 3D printing technology has the potential to serve as an intuitive and convenient surgical planning tool for interventionalists, particularly in the context of bioprosthesis selection. By leveraging this technology, the success rate of TPVR can be significantly increased while also reducing the incidence of postoperative complications.

We utilized an enlarged nRVOT 3D-printed model to assess the feasibility of using the PT-Valve for TPVR and its unique advantages in this patient population. Following the implantation of the PT-Valve into the model, we observed that stable anchorage was achievable, even in cases with large nRVOT sizes and pyramidal RV–PA conduits. This result can be attributed to the self-expanding nature of the PT-Valve, which exerts minimal radial forces compared to balloon-expandable valves [27,28]. Additionally, the symmetrical design of the prosthesis allows for sufficient contact area between the dilated MPA and the nRVOT. The corolla at both ends of the bioprosthesis can be expanded to varying degrees and anchored on both sides of the RV–PA conduit rather than relying solely on radial support at the stent’s cylindrical site. During the preoperative phase, we did not use cardiac computed tomography to accurately measure the diameter of each plane of the RV–PA conduit because of the lack of a clearly defined landing zone for TPVR within the RVOT or MPA. Instead, it was crucial to measure the maximum dimensions of the landing zone as predetermined. Moreover, our model simulations demonstrated that utilizing multiple planes of the conduit offered improved support for the expanded corolla segments of the stent at both ends of the selected bioprosthesis. Even in cases of enlarged nRVOT, the stent demonstrated a good fit to the conduit’s wall, ensuring stent stability. Patients 1 and 2 in our study both had enlarged nRVOTs and pyramidal RV–PA conduits. However, with the guidance of the 3D printing model and the selection of optimal landing zones, the corolla portion of the stent demonstrated uniform expansion to different degrees on both sides of the conduits. This approach effectively ensured valve stability. Additionally, the large corolla at both ends of the valve, designed to prevent regurgitation, proved effective in preventing the occurrence of PVL. During the postoperative follow-up, it was confirmed that the bioprosthesis remained in the optimal anchoring position, as determined by preoperative 3D printing, in eight patients. There were no cases of valve displacement or embolization, and only two of these patients experienced grade 1 (trace) PVL, which did not necessitate a secondary intervention.

The PT-valve annular dimension is intentionally smaller than the diameter of the stent ends, which helps to prevent compression of the valve in the center of the stent and reduces the risk of valve distortion. This feature ensures an effective transvalvular gradient and promotes the long-term durability of the leaflets [29,30]. Current guidelines recommend the determination of the coronary trajectory before carrying out TPVR in patients with repaired TOF. Additionally, the coronary compression test (class Ib) is also recommended in these cases [17]. Following an evaluation of the model, it was observed that the cylindrical part of PT-Valve, particularly in a pyramidal-shaped conduit, achieved stress-free contact with the conduit’s wall. This design minimizes the risk of coronary compression and eliminates the need for repeated balloon inflation tests before implanting the valve. This reduction in procedure and radiation time is beneficial. In our study, only patient 6 underwent a balloon inflation test. This test was necessary because the narrowest plane of the conduit in this patient was the pulmonary annulus, which had a diameter of 26 mm, matching the intended size of the prosthesis annulus. In this case, there was a potential risk of pressure on the annulus and surrounding tissues, including the left coronary artery located beneath the pulmonary annulus, making the test necessary to assess any compression risks. However, after conducting repeated tests, we did not observe coronary compression in this case, and the procedure proceeded smoothly. Given the complexity and variability of the structure of the RVOT, it is necessary to have multiple sizes of frames and valves available for the implanted bioprosthesis. The PT-Valve currently offers a combination of five different frame sizes (28/32/36/40/44 mm) and three different valve sizes (20/23/26 mm). This spectrum of sizes provides flexibility in selecting the appropriate prosthesis sizes for implantation in patients with varying degrees of enlargement undergoing nRVOT. For instance, in our study, we successfully implanted a 26 mm valve with both 44 mm and 36 mm frames in nRVOTs of different sizes.

## 5. Conclusions

We successfully demonstrated the safety, efficacy, and feasibility of implanting PT-Valves in patients with an enlarged nRVOT for the treatment of severe PR. The bioprosthetic function and morphology were well maintained, as evidenced by the postoperative 30-day, 6-month, and 1-year follow-up data. The utilization of preoperative 3D-printing guidance played a crucial role in exploring the properties of the new device, selecting the appropriate size of bioprosthesis for patients with a complex enlarged nRVOT, visualizing the optimal landing position in the conduits, and predicting the risk of complications.

## Figures and Tables

**Table 1:** Patient characteristics.

Characteristics	Patient 1	Patient 2	Patient 3	Patient 4	Patient 5	Patient 6	Patient 7	Patient 8
Age, years/sex	48/F	31/F	17/M	13/F	17/F	52/M	12/M	17/M
Weight (kg)	65	61	70	55	75	50	56	56
Height (cm)	155	168	175	154	164	168	150	170
Symptoms	Chest tightness, edema,	Palpitation, edema	Edema, dyspnea	Palpitation, edema, dyspnea	Chest tightness, edema	Chest tightness, edema, dyspnea	Edema, dyspnea	Chest tightness, edema
QRS duration (ms)	116	127	119	109	147	134	131	139
6-MWT (m)	290	350	390	420	310	300	390	340
NYHA functional class	IV	III	III	II	III	IV	II	II
Diagnosis	PR, rPA(TOF)	PR, rTOF	PR, rPA(TOF)	PR, rTOF + rVSD	PR, rTOF	PR, rTOF	PR, rTOF	PR, rTOF + AVR
Atrial fibrillation/atrial flutter	Yes	Yes	No	No	No	Yes	No	No
Peak O2	15	19	24	23	26	14	19	21
NT-proBNP	1961	1672	961	541	1316	1782	782	1542
RVOT repair age, year	22	22	1	1	3	30	1	2
RVOT type	nRVOT	TAP	nRVOT	TAP	TAP	TAP	TAP	TAP

Values are presented as *n* (%) or median (25th, 75th percentile). 6-MWT—6-min Walking Test; AVR—aortic valve replacement; NT-proBNP—N-terminal pro-B-type natriuretic peptide; NYHA—New York Heart Association; PR—pulmonary regurgitation; rPA—repaired pulmonary atresia; rTOF—repaired Tetralogy of Fallot; RVOT—right ventricular outflow tract; rVSD—residual ventricular septal defect; TAP—transannular patch; TOF—tetralogy of Fallot.

**Table 2:** Baseline imaging analysis and three-dimensional printing guidance.

Echocardiographic Parameters	Patient 1	Patient 2	Patient 3	Patient 4	Patient 5	Patient 6	Patient 7	Patient 8
PR severity grade	4+	4+	4+	4+	4+	4+	4+	4+
Peak transpulmonary valve gradient	21	7	11	13	15	14	9	9
TR severity grade	3+	3+	2+	2+	1+	0	2+	1+
TASPE (mm)	15	16	17	16	18	14	15	16
RV–FAC (%)	34	33	32	34	36	32	34	31
Computed tomography parameters								
RV–PA conduit length (mm)	57	63	48	56	55	46	43	52
nRVOT diameter (mm)	46	42	35	30	30	33	31	33
The narrowest plane/diameter (mm)	Mid MPA/34	PA/ 37	Distal MPA/23	Distal MPA/24	Mid MPA/24	PA/ 26	Distal MPA/22	Mid MPA/23
MRI parameters								
PR fraction (%)	55	52	48	41	40	46	59	54
RVEDVI (ml/m2)	189	176	163	158	165	156	171	156
RVEF (%)	26	21	18	26	24	19	27	21
3D Printing Guidance								
Valve size (mm)	44–26	44–26	36–26	32–23	32–23	32–23	36–26	36–26
Proximal landing zone	RVOT	PA	RVOT	PA	PA	RVOT	RVOT	RVOT
Distal landing zone	Distal MPA	MPA sinus	Mid MPA	MPA sinus	Mid MPA	Mid MPA	Mid MPA	Mid MPA

Values are presented as *n* (%) or median (25th, 75th percentile). The valve sizes are shown as diameter 2–diameter 1, as illustrated in Figure 1. 3D—3-dimensional; MPA—main pulmonary artery; MRI: magnetic resonance imaging; nRVOT—native right ventricular outflow tract; PA—pulmonary annulus; PR—pulmonary regurgitation; RVEDVI—right ventricular end-diastolic volume index; RVEF—right ventricular ejection fraction; RV–FAC—right ventricle–fraction of area change; RVOT: right ventricular outflow tract; RV–PA—right ventricle–pulmonary artery; TAPSE—tricuspid annular plane systolic excursion; TR—tricuspid regurgitation.

**Table 3:** Perioperative and in-hospital outcomes.

Perioperative Outcomes		Patient 1	Patient 2	Patient 3	Patient 4	Patient 5	Patient 6	Patient 7	Patient 8
Right Heart Catheterization Parameters									
Pre-/Post-devices	PASP (mmHg)	32/36	36/39	39/35	34/37	36/38	35/37	32/35	38/37
	PADP (mmHg)	6/11	5/12	3/11	4/9	3/8	7/12	2/7	3/9
	mRAP (mmHg)	10/4	9/7	6/5	5/4	7/4	8/5	8/6	10/8
	RVEDP (mmHg)	8/5	7/4	11/6	10/4	9/5	6/3	8/6	6/5
	PA–RV gradient (mmHg)	10/6	8/2	−4/−2	−1/−3	9/6	2/−2	−3/−5	2/−1
Intraoperative, post-devices TEE									
Residual PR		None	None	Trace	None	None	None	Trace	None
Peak transpulmonary valve gradient		11	10	6	8	9	8	10	7
Operative outcomes									
Procedure time (minutes)		52	57	68	62	58	63	71	63
Fluoroscopy time (minutes)		26	14	16	17	21	15	24	19
Balloon inflation test		No	No	No	No	No	No	Yes	No
Coronary compression		No	No	No	No	No	No	No	No
In-hospital outcomes									
ICU time (days)		2	1	2	1	1	2	1	1
Postoperative hospitalization time (days)		5	4	5	4	4	5	4	4
Procedural success		Yes	Yes	Yes	Yes	Yes	Yes	Yes	Yes
Device-related adverse events		None	None	None	None	None	None	None	None
Complications		None	None	Hemoptysis	None	None	None	Hemoptysis	None

Values are presented as *n* (%) or median (25th, 75th percentile). ICU—intensive care unit; mRAP—mean right atrial pressure; PADP—pulmonary artery diastolic pressure; PA-RV—pulmonary artery–right ventricle; PASP—pulmonary artery systolic pressure; PR—pulmonary regurgitation; RVEDP—right ventricular end-diastolic pressure; TEE—transesophageal echocardiography.

**Table 4:** Baseline to 1-year follow-up.

		Baseline	30 Days		6 Months		1 Year	
Clinical Characteristic			Results	*p*-Value	Results	*p*-Value	Results	*p*-Value
Peak O2		20 (15, 14)	21 (17, 26)	0.466	25 (22, 29)	0.014	29 (24, 33)	0.002
6-MWT (m)		345 (300, 390)	420 (390, 460)	0.003	445 (415,480)	<0.001	460 (420, 495)	<0.001
QRS duration (ms)		129 (116, 139)	110 (101, 139)	0.072	114 (96, 142)	0.053	112 (98, 138)	0.106
Echocardiographic parameters								
PR severity	None, *n* (%)	0 (0.0)	8 (100.0%)	<0.001	7 (87.5%)	<0.001	6 (75.0%)	<0.001
	Trace, *n* (%)	0 (0.0)	0 (0.0)	——	1 (12.5%)	0.231	2 (25.0%)	<0.001
	Mild, *n* (%)	0 (0.0)	0 (0.0)	——	0 (0.0)	——	0 (0.0)	——
	Moderate, *n* (%)	0 (0.0)	0 (0.0)	——	0 (0.0)	——	0 (0.0)	——
	Severe, *n* (%)	8 (100.0%)	0 (0.0)	<0.001	0 (0.0)	<0.001	0 (0.0)	<0.001
Peak transpulmonary valve gradient (mmHg)		13.5 (9, 14)	9 (8, 15)	0.183	10(8, 11)	0.148	11 (9, 13)	0.550
TASPE (mm)		16 (15, 17)	16 (14, 17)	0.998	16 (15, 17)	0.364	17 (16, 19)	0.023
RV–FAC (%)		33.5 (32, 34)	34 (33, 36)	0.138	37 (34, 39)	0.003	38 (36,41)	<0.001
MRI parameters								
PR fraction (%)		50 (41, 55)	——	——	2.5 (2, 3.5)	<0.001	1.5 (1, 2)	<0.001
RVEDVI (ml/m2)		164 (156, 176)	——	——	137 (126, 168)	0.004	119 (107, 148)	<0.001
RVEF (%)		22.5 (19, 26)	——	——	31 (28, 34)	<0.001	33 (31, 39)	<0.001

Values are presented as N (%) or median (25th, 75th percentile). 6-MWT—6-min Walking Test; MRI—magnetic resonance imaging; PR—pulmonary regurgitation; RVEDVI—right ventricular end-diastolic volume index; RVEF—right ventricular ejection fraction; RV-FAC—right ventricle fraction of area change; TAPSE—tricuspid annular plane systolic excursion.

## Data Availability

Data were uploaded as suggested by Data Availability Statements in the section, “MDPI Research Data Policies”.
